# Warmblood fragile foal syndrome causative single nucleotide polymorphism frequency in horses in Ireland

**DOI:** 10.1186/s13620-021-00206-1

**Published:** 2021-10-18

**Authors:** Áine Rowe, Sharon Flanagan, Gerald Barry, Lisa M. Katz, Elizabeth A. Lane, Vivienne Duggan

**Affiliations:** 1 Veterinary Sciences Centre, School of Agriculture, Food Science and Veterinary Medicine, University College Dublin, Belfield, Dublin 4, Ireland; 2 Animal Health Division, Department of Agriculture, Food and the Marine, Backweston Campus, Celbridge, Co. Kildare W23 X3PH Ireland

**Keywords:** Warmblood fragile foal syndrome, PLOD1, Horse, Skin, EDLS, Hereditary, Genetic testing

## Abstract

**Background:**

Warmblood Fragile Foal Syndrome (WFFS) is an autosomal recessive disorder caused by a mutation in the procollagen-lysine, 2-oxoglutarate 5-dioxygenase 1 (*PLOD1*) gene. Homozygosity for the mutation results in defective collagen synthesis which clinically manifests as the birth of non viable or still born foals with abnormally fragile skin. While the mutation has been identified in non Warmblood breeds including the Thoroughbred, to date all homozygous clinically affected cases reported in the scientific literature are Warmblood foals. The objective of this study was to investigate the carrier frequency of the mutation in the Thoroughbred and sport horse populations in Ireland.

**Methods:**

A test was developed at the UCD School of Veterinary Medicine using real-time PCR to amplify the PLOD1 gene c.2032G > A variant. A subset of the samples was also submitted to an external laboratory with a licensed commercial WFFS genetic test.

**Results:**

Warmblood Fragile Foal Syndrome genotyping was performed on hair samples from 469 horses representing 6 different breeds. Six of 303 (1.98%) sport horses tested and three of 109 (2.75%) Thoroughbreds tested were heterozygous for the WFFS polymorphism (N/WFFS). The WFFS polymorphism was not identified in the Standardbred, Cob, Connemara, or other pony breeds.

**Conclusions:**

The study identified a low frequency of the WFFS causative mutation in sport horses and Thoroughbreds in Ireland, highlighting the importance of WFFS genetic testing in order to identify phenotypically normal heterozygous carriers and to prevent the birth of nonviable foals.

## Background

Ehlers Danlos syndrome (EDS) describes a group of heritable connective tissue disorders affecting humans, characterized by abnormal collagen biosynthesis and manifesting as hyperextensible, abnormally fragile skin, joint hypermobility, and vessel and tissue fragility [1, 2]. Ehlers Danlos like syndromes (EDLS) have been observed in several breeds of horses, including Draught horses [3], Thoroughbreds [4], Arabians [5], Warmbloods [6–8] and Quarter Horses [9].

To date, two gene defects causing EDLS phenotypes in horses have been discovered, hereditary equine regional dermal asthenia (HERDA), a degenerative inherited autosomal recessive skin disorder occurring in Quarter Horses and related breeds [10], and Warmblood fragile foal syndrome (WFFS), a lethal congenital autosomal recessive disorder, which to date has only been reported in Warmblood foals [11, 12]; however, the WFFS causative mutation has been identified in Thoroughbreds [13, 14], Draft Horses, Paint Horses, Quarter Horses [15] and in the American Sport Pony [16].

WFFS is caused by a point mutation (c.2032G > A, p.Gly678Arg) in the equine procollagen lysine, 2-oxoglutarate 5 -dioxygenase 1 (PLOD1) gene which encodes lysyl hydroxylase [12]. In humans, this enzyme catalyzes the hydroxylation of lysyl residues in collagen sequences, which then serve as binding sites for galactose/glucosyl-galactose units, allowing for intra and intermolecular crosslinking of collagen fibrils [17]. Deficiency of lysyl hydroxylase due to mutated PLOD1 in humans results in mechanical instability of affected connective tissue [18, 19].

The birth of non viable or still born foals with skin lesions is the most characteristic clinical manifestation of WFFS [13]. The forelimbs, neck and abdomen are most commonly affected [11, 13, 20]. In some cases, foals have an open abdomen and small intestinal eventration [11, 13, 20]. Flexural deformities, deformities of the spinal canal and perforating lesions of the aorta have also been described [11, 13]. The homozygous WFFS genotype is incompatible with extra uterine life and foals are born dead, die or require euthanasia on humane grounds shortly after birth [13]. There have been no abnormalities associated with the heterozygous genotype reported in the literature.

The source of the WFFS mutation has not been identified; therefore, it remains unknown in which horse breeds the mutation may occur today. The English Thoroughbred stallion Dark Ronald (1905–1928), initially suggested to be the source of the allele based on pedigree analysis, was recently found to be homozygous wildtype at the WFFS causative variant position [21]. Recent research demonstrated that the Arabian stallion Bairactar Or. Ar (1813), also hypothesized to be the origin of the WFFS allele, was not the source of the mutation and did not support the hypothesis that the mutation originated in the Arabian breed [16]. Pedigree analysis for genotyped genetic carriers across multiple generations resulted in the identification of a Hanoverian stallion born in 1861 as the most recent common ancestor of investigated carriers [20] but it has been hypothesized that the origin of the mutation can be traced further back to the Thoroughbred breed, which has contributed significantly to today’s Warmblood population [22].

A number of studies have looked at the carrier frequency of the gene in horse populations around the world. A carrier frequency of 11% was reported in a population of Warmbloods in the United States [12] and in Brazil [23]. Similar carrier frequencies have been reported in Warmbloods in Europe [11, 13]. More recently, a higher WFFS allele carrier frequency of 17% in the Hanoverian and Danish Warmblood breeds was described [16], while Metzger et al. reported a carrier frequency of 28% in Hanoverian horses in Germany [20]. In a further study, 2.4% of Thoroughbreds in the United States (*n* = 716) were identified as carriers of the allele [14]. The prevalence of the WFFS allele in Thoroughbreds in Europe is currently unknown.

A number of horse and pony breeds are native to Ireland including the Connemara pony, the Kerry Bog Pony, the Irish Cob and the Irish Draft Horse. The Irish Sport Horse (ISH) is a composite of the Irish Draft horse and the Thoroughbred breeds, although more recently, introduction of continental Warm blood lines has become more common with the potential unrecognized dissemination of the WFFS allele in the Irish equine population. In general, in Ireland, non-Thoroughbred horses are considered sport horses, albeit all may not be registered as Irish Sport Horses. The prevalence of the WFFS mutation in Irish horse populations is unknown.

The aim of this study was to determine the carrier frequency of the WFFS allele in the Irish Thoroughbred and sport horse populations. We hypothesized that the prevalence of the allele would be lower than that reported in mainland Europe. We also aimed to estimate the potential impact of WFFS both on the health of Irish horses and on the Irish equine industry.

## Methods

The sample size to determine the frequency of heterozygotes for WFFS in Ireland was calculated using OpenEpi software (version 3.0.1). To date, all prevalence studies have investigated the prevalence of the WFFS allele in Warmbloods or Thoroughbreds and report a prevalence of approximately 11 and 2.4% respectively [11, 13, 14]. While the mutation has been identified in other breeds, there is currently no data on prevalence in breeds other than the Warmblood and Thoroughbred available. The Irish Sport Horse is a composite of Irish Draft horses and Thoroughbreds, with the recent addition of Warm blood lines. We therefore estimated a lower prevalence than that reported in Warmbloods in Europe.

With 137,000 sport horses registered in Ireland [24], an estimated prevalence of heterozygotes for WFFS of 5%, and a 5% margin of error for the anticipated frequency, a minimal sample size of 151 sport horses was required, providing a 99% confidence level. Similarly, with 37,000 Thoroughbred horses registered in Ireland [25], an estimated prevalence of heterozygotes for WFFS of 2.4% [14] and a 5% margin of error for the anticipated frequency, a minimal sample size of 102 Thoroughbreds was required, with a 99% confidence level.

Hair root samples were collected from young (3-year-old) sport horses attending national sport horse competition qualifier events in Munster, Leinster, Connacht and Ulster. Hair samples were also collected from all breeds of horses and ponies treated at the UCD Veterinary Hospital and from horses on stud farms and equestrian centres in the Leinster region. Veterinarians from multiple locations in Ireland were requested to submit hair samples from clients’ horses. All samples were taken with owner consent under a confidentiality agreement to ensure the anonymity of horses, owners and establishments.

A real-time PCR assay, developed in-house was used to differentiate between carriers, homozygous wild type and homozygous mutant samples. The full protocol is published on protocols.io entitled: ‘PCR protocol to identify an equine mutation associated with Warmblood Fragile Foal Syndrome (WFFS)’ and is available at dx.doi.org/10.17504/protocols.io.bw4fpgtn. Briefly, DNA fragments containing the relevant WFFS target sequences and flanking sequence were synthesized and used as controls and to optimise the PCR, ensuring the PCR could distinguish the single nucleotide difference between wild type and mutant. DNA was extracted from horse hair using a Qiagen DNA Blood and Tissue kit and approximately 10 ng/μl of genomic DNA was used in each PCR. The real-time PCR assay was based on a TaqMan SNP Genotyping assay (Thermo Fisher Scientific). Primers were designed to amplify the region that contained the potential PLOD1 mutations and probes were designed to identify either the wild type or the mutated sequence. All sequences for the primers and probes are available at protocols.io as mentioned above. PCR conditions were initially optimised using control plasmids. Following optimization using plasmids, the PCR was optimized by testing various numbers of horse hair during the extraction, varying different steps of the extraction protocol to ensure optimal yield and then varying the input amount of DNA for the PCR to identify the minimum concentration of DNA required to produce a consistent result. Different concentrations of samples were tested and 10 ng/μl was found to be optimal. Samples were run in duplicate each time and repeated at least three times to confirm the robustness of the assay, with a consistent result achieved each time. Samples were also submitted in an anonymous format to an external laboratory with a licensed commercial WFFS genetic test and results were taken as current ‘gold standard’. Samples with known genotype were assessed blind by PCR to ensure consistency of result.

Warmblood Fragile Foal Syndrome carrier frequencies were calculated using Excel (Microsoft Office).

## Results

Hair root samples (*n* = 469) were collected from 94 young (3-year-old) sport horses, from 158 horses and ponies treated at the UCD Veterinary Hospital and from 160 horses on stud farms and equestrian centres in the Leinster region. Private veterinarians from multiple locations in Ireland submitted 57 hair samples. Samples represented 6 different breeds and a range of age, breed and sex, as summarized in Table 1.

**Table 1:** Breeds and sex of horses on which WFFS genotyping was performed

Breed	Mares	Geldings	Colts	Stallions	Sex Unknown	Total Number
**Sport Horse**	**140**	**120**	**16**	**9**	**18**	**303**
N/WFFS	4 (4.28%)	1 (1.66%)	1 (6.25%)	0	0	**6 (1.98%)**
N/N	136 (97.14%)	119 (99.16%)	15 (93.75%)	9 (100%)	18 (100%)	**297 (98%)**
**Thoroughbred**	**78**	**16**	**6**	**7**	**2**	**109**
N/WFFS	3 (3.84%)	0	0	0	0	**3 (2.75%)**
N/N	75 (96.15%)	16 (100%)	6 (100%)	7 (100%)	1 (100%)	**105 (97.22%)**
**Other**	**27**	**29**	**1**	**0**	**0**	**57**
N/WFFS	0	0	0	0	0	**0**
N/N	27 (100%)	29 (100%)	1	0	0	**57 (100%)**
**Total**	**245**	**165**	**23**	**16**	**20**	**469**
N/WFFS	7 (2.85%)	1 (0.01%)	1 (0.41%)	0	0	**9 (1.9%)**
N/N	238 (97.14%)	164 (99%)	22 (95.6%)	16 (100%)	20 (100%)	**460 (98.08%)**

*N/WFFS* heterozygous carrier, *N/N* homozygous non-carrier

WFFS genotyping was performed on all samples. Of the samples tested, 376 were tested by the PCR only; 74 tested by the commercial test only and 19 tested by both methods.

Of the 19 samples tested using both tests, 18 of the tests results concurred. Of the 18 samples, 15 were homozygous for the wildtype allele on both tests and 3 were heterozygous for the WFFS polymorphism on both tests. One sample was heterozygous for WFFS on the commercial test with an inconclusive result on the in-house PCR test; however, a limited number of hair follicles were available for testing using the in-house PCR method. It was not possible to obtain another hair sample from the horse in question for repeat testing. This horse was considered positive for the purposes of this study.

In total, 9 of 469 horses (1.9%) were identified as heterozygous for the WFFS polymorphism (N/WFFS) (Table 2). The carrier horses all originated from different herds and from the east and west of the country. Seven of the 9 horses were female, one was an intact male and one was a gelding. The ages of the carrier horses ranged from 3 to 10 years old. The 460 homozygous wild type horses (N/N) consisted of 238 females, 38 intact males, 163 geldings and 20 of unknown gender. Ages of the non-carrier horses ranged from 3 to 30 years old.

**Table 2:** Horses heterozygous positive for the WFFS polymorphism

	Breed	Sex	Age (years)
1	Irish Sport Horse	Female	3
2	Irish Sport Horse	Female	3
3	Irish Sport Horse	Female	4
4	Sport Horse (Warmblood)	Female (broodmare)	4
5	Irish Sport Horse	Male (colt)	3
6	Sport horse	Male (gelding)	12
7	Thoroughbred	Female	7
8	Thoroughbred	Female	6
9	Thoroughbred	Female	Unknown

Six of 303 (1.98%) sport horses tested were identified as heterozygous for the WFFS polymorphism (N/WFFS). The remaining 297 sport horses (98.01%) were homozygous for the wild-type allele (N/N). Of the heterozygous sport horse carriers, four were female, one was a colt and the other was a gelding. They originated from different parts (east and west) of the country. The four female sport horses and the colt sport horse carriers were 3–4 years old and had not yet been used for breeding purposes. The remaining sport horse carrier was a 10 year old gelding.

Three of 109 (2.75%) Thoroughbreds tested were heterozygous for the WFFS polymorphism (N/WFFS). The other 106 (97.2%) were homozygous for the wild type allele (N/N). The 3 heterozygous Thoroughbred carriers were all adult broodmares aged between 6 and 10 years old. They originated from 3 different unrelated yards or farms in the east of the country. No male Thoroughbred carrier horses were detected. The WFFS polymorphism was not identified in other breeds.

## Discussion

### The test

This study utilised a commercially available licensed test and a newly developed in-house rapid real time PCR test, based on the same patent data, for identification of the WFFS allele. Nineteen horses were tested with both tests. Identical results were obtained in 18 of these horses. The final sample was positive with the commercial test, but the in-house test had insufficient hair to obtain a clear result and the tested horse could not be resampled. However, the concurrency of the 18 identical tests, both positive and negative, renders the in-house test accurate and reliable, as long as sufficient genetic material is available.

### Frequency

This study identified a low frequency (1.91%) of the WFFS allele in the horse population in Ireland. The carrier horses originated from individual and unrelated yards and farms throughout the east and in the west of the country. They were young sport horse and Thoroughbreds horses, ranging in age from 3 to 10 years old. While the detection of the allele in sport horses may have been influenced by importation of European stallion semen, the presence of the carrier status in older Thoroughbred mares resident in Ireland supports the presence of the allele in Irish Thoroughbred bloodlines.

### Sport horses

The frequency of the WFFS mutation in the sport horse population tested in this study (1.98%) is low in comparison to the reported frequency (11–15%) in Warmblood breeds in mainland Europe, the United States and Brazil [13, 16, 23] While one of the sport horse carriers in this study was reported to be of Warmblood pedigree, most sport horses tested were reported to be ‘Irish Sport Horses’. The low frequency of the WFFS allele in sport horses in Ireland may be due to the influence of the traditional Irish Draught and Thoroughbred breeds on the modern Irish Sport Horse. The fact that five of the six heterozygous sport horse carriers were under the age of 4 years may be reflective of the recent increased use in Ireland of imported semen from European Warmblood stallions.

This study was not designed to look at pedigree information and there was insufficient data to analyze the allele frequency in horses of known Warmblood or Irish Sport Horse lineage. Further investigation into the frequency of the mutation in Warmbloods and sport horse lines in Ireland is required.

### Thoroughbreds

This study indicates that there is a low frequency of the WFFS allele in the Thoroughbred population in Ireland (2.75%). This is similar to the frequency (2.4%) reported for Thoroughbreds (*n* = 716) in the United States [14]. More recently, genotyping of 146 Thoroughbreds from Poland revealed all to be homozygous for the wild type allele (N/N); however, this was a regionally restricted sample [16].

The Thoroughbreds sampled as part of the current study were a convenience sample that was not nationally distributed. All Thoroughbreds sampled were in the Leinster region and a large number of broodmares and stallions from the same farms were sampled. A more regionally distributed sample would allow for a more accurate representation of the frequency of the allele in Thoroughbreds in Ireland. However, the three carrier Thoroughbred mares that were identified originated in unrelated yards and farms across the sampled region. There was insufficient pedigree data available to draw any inference about affected blood lines in Irish Thoroughbred horses.

### Other breeds

In this study the PLOD1 variant was not detected in any Cobs, Standardbreds, Connemara or other Pony breeds tested, although the number of horses tested belonging to these breeds was too low to draw conclusions on breed prevalence. The WFFS allele has been reported in the American Sport Pony [16], Quarter Horse [15] and Draft Horse [15], suggesting that further investigation is required to more accurately determine the prevalence PLOD1 variant in these breeds.

### Impact in Ireland

The carrier animals in this study were predominantly intact females, 4 of which were currently being used for breeding purposes. The speed of dissemination of an allele to the offspring of a carrier female is much less than for a carrier male. Under natural breeding conditions, the female may pass on the allele to one foal every 2 years, on average. However, the carrier male, may pass the allele on to 50% of the pregnancies in the mares he covers in one season. In this study only 1 of 39 intact male horses was a carrier and that was a 3 year old Irish Sport Horse colt which had not yet been used for breeding. However, the stallions tested in this study were from limited locations and herds and do not represent the greater population of stallions in Ireland. In addition, this study did not set out to determine the prevalence of the allele in stallions in Ireland, and has insufficient power and pedigree information to do so.

Identification of carrier stallions in Ireland is key to determining the potential impact of this disease on the Irish equine population. Notwithstanding this, increased use of European Warmblood stallions in the Irish sport horse industry in recent years may pose additional risks for breeders. Therefore, breeders and veterinarians should be aware of the risk of unintentional allele dissemination by the breeding of unknown WFFS carriers and of the potentially fatal outcome associated with the breeding of two WFFS heterozygous carriers.

### Clinical disease

Reported clinical disease associated with the homozygous status is confined to Warmblood foals. No Thoroughbred foal affected with WFFS has been reported in the literature. One non-viable Thoroughbred foal submitted for necropsy was found to be heterozygous for the WFFS allele [13], but this foal died of non WFFS causes. To the authors’ knowledge, no clinical case of WFFS in Ireland has been reported in the literature or by any neonatal specialist in veterinary practice in Ireland (personal communication with neonatal specialist veterinarians).

The lack of clinical evidence of this disease in Irish neonatal foals may be due to the low WFFS carrier frequency identified in this study, in particular in breeding females and intact males. But it is also possible that cases of WFFS are not diagnosed due to a lack of awareness of the disorder on the part of breeders and veterinarians. Awareness of the disorder has only increased in recent years, since the publication of the first case report and the introduction of mandatory WFFS genetic testing by some Warmblood breed societies [11].

Veterinarians and breeders should be aware that not all affected foals have detectable skin lesions at birth and some have minimal skin lesions, for example lesions restricted to the tail or perineal region [13]. This may lead to a small proportion of WFFS cases being misdiagnosed. In one study, amongst 15 cases in which WFSS was suspected, 14 were homozygous for the WFFS allele and none of the fetuses submitted for necropsy without a suspicion of the disorder were found to be homozygous for the allele. Therefore, while the vast majority of WFFS affected foals have obvious morphological abnormalities, the lack of skin defects in non viable foals should not preclude WFFS genetic testing.

### Fetal viability

In countries where higher frequencies of the mutant allele are reported in the equine population than in this study, the incidence of WFFS has been considered less than expected, prompting the hypothesis that homozygosity for the WFFS allele may result in pregnancy loss. Ehlers Danlos syndrome in humans has been reported to cause premature rupture of fetal membranes, which contain large amounts of collagen [26]. To study this, Aurich et al. described the pathological abnormalities associated with 14 WFFS homozygous fetuses and concluded that rather than late stage gestational losses, the birth of nonviable full term foals was the most common manifestation of the disorder, although abnormalities of fetal development earlier in pregnancy could not be excluded [13]. More recently Metzger et al. supported the suggestion that WFFS losses were primarily caused by the birth of nonviable foals, finding no significant correlation between the WFFS variant and estimated breeding values for embryonic survival in progeny of 195 Hanoverian stallions [20].

None of the 4 Thoroughbred or sport horse carrier broodmares in this study had any significant reported history of reproductive loss. If abnormalities of fetal development are a feature of this disease, WFFS genetic testing of non-viable foals may allow for a more accurate interpretation of the prevalence of the mutation in the population.

### Genetic testing

In order to establish the true impact of this disease in Ireland more extensive genetic testing of breeding stallions and still born or unviable foals is required. However, there is a reluctance among many breeders, across various equine breeds to engage with genetic testing. In this study, in spite of arranging approval for hair sampling in advance from event organisers, through the veterinary advisor, and only sampling horses for which the owners provided consent, we experienced hesitance among breeders to engage with the study. However, the results of this study highlight the importance of WFFS genetic testing in order to prevent the inadvertent mating of carrier sire and dam and the birth of nonviable foals.

The aim of identifying carriers of the allele is not to attempt to remove the allele from the population. Although, theories abound on clinical abnormalities that may be present in carriers of this allele, there are no studies that have confirmed collagen defective conditions in these carrier animals. One recent study identified no association between the WFFS allele and catastrophic breakdown in Thoroughbreds [13]. Indeed, significant correlations of the WFFS variant with breeding values for performance traits such as gaits and rideability were recently reported by Metzger et al. [20].

The aim of the identification of carriers of the allele is to facilitate informed breeding choices.

Knowledge of the WFFS status of mares allows the avoidance of the mating of two WWFS carriers while also allowing WFFS heterozygous breeding animals to remain in the gene pool, propagating desirable traits and maintaining maximum genetic diversity. The WFFS status of stallions is reported by Warmblood breed societies in Europe and mandatory testing of stallions has recently been introduced in some European countries, in order to facilitate informed breeding decisions. Horse Sport Ireland began compulsory WFFS testing of stallions undergoing HSI studbook inspection 2 years ago and also offer free testing for Sport Horse foals on a voluntary basis.

### Limitations

The population of Thoroughbreds sampled as part of this study was regionally restricted for convenience reasons to the Leinster area. Additionally, many of the Thoroughbred broodmares and stallions sampled were from the same farm. A broader based sample would allow for more accurate determination of the national prevalence of the mutation in this breed.

The majority of Sport horses sampled were young horses or horses not used for breeding purposes. Further sampling specifically of sport horse and Thoroughbred broodmares and stallions would better reflect the current potential reproductive significance of the disorder in Ireland. However, the large sample of young sport horses is representative of current breeding practices in Ireland and of the future gene pool.

Further information on the pedigree of the horses in this study would have provided information on the influence of particular Warmblood, sport horse and Thoroughbred lines on the prevalence of the mutation in Ireland. That was not the aim of this study but would be a useful future study. The frequency of the WFFS allele in European Warmblood horses in Ireland remains unknown.

## Conclusions

In conclusion, this study identified a low frequency of the WFFS causative mutation in the PLOD1 gene in sport horses (1.98%) and Thoroughbreds (2.75%) in Ireland. The low frequency in the sport horse population is likely a reflection of traditional breeding, which is influenced by the Thoroughbred and Irish Draft horse lines.

Given the low frequency of the WFFS allele in horses in Ireland, and the apparent lack of clinical evidence of the condition, the current economic impact of the disorder on the Irish equine industry appears relatively low. However, the predominance of young carrier animals with breeding potential in this study could herald an increase in the prevalence of this condition in the national herd in future years, in particular if carrier males remain intact and are used for breeding.

Considerable economic losses are sustained by the individual breeder in the event of the birth of a WFFS affected foal. Knowledge of sire and dam WFFS status allows informed breeding decisions to be made while maintaining genetic diversity.

Available genetic testing should be utilized with the goal of improving the health and welfare of horses and safeguarding the equine industry. Veterinarians should be proactive in this area. The reporting of suspected genetic disease and submission of DNA samples for testing will increase our knowledge about the disorder, which can then be translated into best practices for breeding, thus improving the overall health of the equine population.

## Data Availability

The datasets generated during the current study are not publicly available in order to maintain confidentiality of the horse owners involved in the study.

## Abbreviations

EDS: Ehlers Danlos Syndrome
EDLS: Ehlers Danlos Like Syndrome
HERDA: Hereditary Equine Regional Dermal Asthenia
WFFS: Warmblood Fragile Foal Syndrome
